# The Representational Challenge of Integration and Interoperability in Transformed Health Ecosystems

**DOI:** 10.3390/jpm15010004

**Published:** 2024-12-25

**Authors:** Bernd Blobel, Frank Oemig, Pekka Ruotsalainen, Mathias Brochhausen, Kevin W. Sexton, Mauro Giacomini

**Affiliations:** 1 Medical Faculty, University of Regensburg, 93053 Regensburg, Germany; 2 First Medical Faculty, Charles University Prague, 12108 Prague 2, Czech Republic; 3 Department of Informatics, Bioengineering, Robotics and System Engineering, University of Genoa, 16145 Genoa, Italy; mauro.giacomini@unige.it; 4 Faculty European Campus Rottal-Inn, Deggendorf Institute of Technology, 84347 Pfarrkirchen, Germany; 5 IT Healthcare Consulting, 45472 Muelheim, Germany; frank@oemig.de; 6 IHE Germany, 10117 Berlin, Germany; 7 Faculty of Information Technology and Communication Sciences, Tampere University, 33014 Tampere, Finland; pekka.ruotsalainen@uta.fi; 8 Department of Biomedical Informatics, University of Arkansas for Medical Sciences, Little Rock, AR 72205, USA; mbrochhausen@uams.edu; 9 Department of Surgery, University of Arkansas for Medical Sciences, Little Rock, AR 72205, USA; kevin.sexton@uams.edu

**Keywords:** health and social care transformation, ecosystems, systems architecture, knowledge representation and management, information modelling, language types, ontologies

## Abstract

**Background/Objectives**: Health and social care systems around the globe are currently undergoing a transformation towards personalized, preventive, predictive, participative precision medicine (5PM), considering the individual health status, conditions, genetic and genomic dispositions, etc., in personal, social, occupational, environmental, and behavioral contexts. This transformation is strongly supported by technologies such as micro- and nanotechnologies, advanced computing, artificial intelligence, edge computing, etc. **Methods**: To enable communication and cooperation between actors from different domains using different methodologies, languages, and ontologies based on different education, experiences, etc., we have to understand the transformed health ecosystem and all its components in terms of structure, function and relationships in the necessary detail, ranging from elementary particles up to the universe. In this way, we advance design and management of the complex and highly dynamic ecosystem from data to knowledge level. The challenge is the consistent, correct, and formalized representation of the transformed health ecosystem from the perspectives of all domains involved, representing and managing them based on related ontologies. The resulting business viewpoint of the real-world ecosystem must be interrelated using the ISO/IEC 21838 Top Level Ontologies standard. Thereafter, the outcome can be transformed into implementable solutions using the ISO/IEC 10746 Open Distributed Processing Reference Model. **Results**: The model and framework for this system-oriented, architecture-centric, ontology-based, policy-driven approach have been developed by the first author and meanwhile standardized as ISO 23903 Interoperability and Integration Reference Architecture. The formal representation of any ecosystem and its development process including examples of practical deployment of the approach, are presented in detail. This includes correct systems and standards integration and interoperability solutions. A special issue newly addressed in the paper is the correct and consistent formal representation **Conclusions**: of all components in the development process, enabling interoperability between and integration of any existing representational artifacts such as models, work products, as well as used terminologies and ontologies. The provided solution is meanwhile mandatory at ISOTC215, CEN/TC251 and many other standards developing organization in health informatics for all projects covering more than just one domain.

## 1. Introduction

Over many years, healthcare systems around the globe have evolved from empiric or phenomenological medicine, locally providing domain-specific general services, through evidence-based medicine, providing domain-specific group-specific services, to person-centered medicine, providing coordinated multiple-domain services to the subject of care, which is also called managed care.

Traditionally, a health professional’s own observations and conclusions (in evidence-based medicine, also observations, conclusions, and solutions from other domain experts available in related databases) have been used, despite the long-term development of anatomy, toxicology, histology, and pathology down to the cellular level. In the 1990s, it became more and more evident that individuals differentiate in their molecular, physiological, and behavioral characteristics, accompanied by different environmental and occupational exposure, etc., but also regarding their individual health history. This led to the development of personalized medicine by providing multiple domain services to the subject of care, including telemedicine. Therefore, the clinically justified individual status and context of the subject of care must be considered and understood.

So far, medicine has been understood as a service for diseased subjects, managed by care professionals. The ongoing healthcare systems transformation aims at personalized, preventive, predictive, participative precision medicine (P5M). It considers individual health status, conditions, genetic and genomic dispositions in personal, social, occupational, environmental, and behavioral contexts, thus turning health and social care from reactive to proactive. Table 1 summarizes the described health transformation. Further aspects, such as presentational challenges, standards, etc., will be discussed later.

In the P5M approach, we cannot consider the health and social care system in isolation, but must incorporate its political, legal, ethical, economic, and ecological framework. We must therefore consider all these domains and their actors as well. In other words, we have to manage the 5PM ecosystem. Here, an ecosystem is defined as a structural and functional unit of ecology where the living organisms interact with each other and the surrounding environment. It is the community of living organisms in conjunction with non-living components of their environment, interacting as a system [1].

The objective addressed in the paper is the use-case-specific formal, correct, and consistent representation of the ecosystem components from the perspective of any domain involved and any viewpoint managed in the system development process. That way, integration and interoperability between the domains, their concept spaces, and actors, but also between models, work products, and used ontologies at the different viewpoints are enabled. A comprehensive overview on managing healthcare transformation towards 5PM is provided in [2]. The paper is structured as follows. First, it introduces challenges, principles, and related standards for understanding and correctly representing ecosystems. Thereby, the need to advance from data to knowledge level of the system design and management is explained. The outcome, a system-oriented, architecture-centric, ontology-based, policy-driven approach and the related standards, especially the ISO 23903 Interoperability and Integration Reference Architecture [3], are introduced. On that basis, comprehensive integration and interoperability between all components of the ecosystem, including the involved domains and their actors, are presented. In Section 5, these general solutions are exemplified for specific use cases from different viewpoints in the development process, as well as for controlling the system’s behavior by appropriate policies.

## 2. Foundations for Designing and Managing Transformed Health and Social Care Ecosystems

It is impossible to represent the highly complex, highly dynamic, multidisciplinary/multi-domain healthcare system by one domain’s terminology or even by using “simple” ICT ontologies. There are approaches for representing multi-domain concepts in a hierarchical set of ontologies. An ontology is a formal explicit specification of a shared conceptualization of a domain of interest, providing an ordering system of entities in a domain and their relations [4]. A concept is a knowledge component the expert community has agreed on. A concept must be uniquely identifiable, and independently accepted by experts and users. To enable consistent communication and cooperation, we have to guarantee that all actors refer to the same real-world component. For this reason, an abstract and generic reference architecture able to represent any perspective or domain of interest for any ecosystem in question is inevitable. A starting point could be an abstract mathematical representation based on universal type theory and universal logics, such as the Barendregt Cube [5], to formally and consistently represent any system in the universe. More details can be found in [6].

For managing systems and systems engineering, a system-theoretical approach is more practical. However, for managing systems in their structure and function, a black box approach just considering the relations of the system and its environment is not sufficient. Instead, we must understand all system elements, their functions and constraints, their composition and decomposition, as well as their internal and external relations by using a white box approach, advancing from an at least partially implicit to a comprehensively explicit representation. Moreover, this must be carried out from the perspectives of all domains and their actors involved in the ecosystem. The granularity level considered depends on the domain experts’ objectives in the context of the actual use case of the business system, and therefore ranges from elementary particles up to the universe. The resulting generic component model (GCM), introduced by the first author in the early nineties of the last century, consists of the following three perspectives or dimensions:

- System’s Architectural Perspective;
- System’s Evolutionary or Development Perspective;
- System’s Domain Perspective.

**Figure 1:**
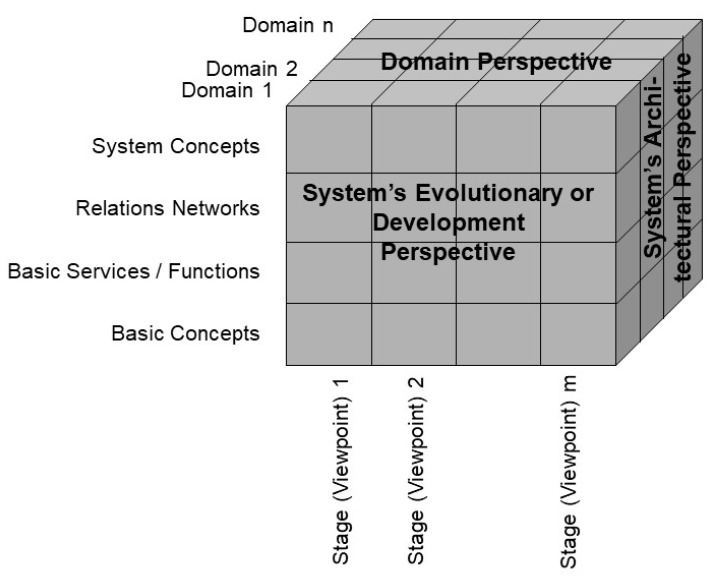
Generic Component Model reference architecture.

Meanwhile, the approach has been standardized as ISO 23903:2021 Interoperability and integration reference architecture—Model and framework [3]. Figure 1 presents the GCM reference architecture.

Regarding the dimension of the system’s evolution or development process from an architectural perspective, there are different standards to manage this process:

- The Object Management Group (OMG) Model Driven Architecture (MDA) [7];
- ISO/IEC 10746 Reference Model for Open Distributed Processing (RM-ODP) [8];
- ISO 23903 Integration and Interoperability Reference Architecture.

The OMG MDA starts with the Computation Independent Model (CIM) as primary model. On that basis, a Platform Independent Model (PIM) is defined, which contains enough information to derive one or more Platform Specific Models (PSMs). The ISO/IEC 10746 Reference Model for Open Distributed Processing provides a methodology for describing and building widely distributed ICT systems and applications. In this, it defines the following viewpoints: enterprise viewpoint, information viewpoint, computational viewpoint, engineering viewpoint, and technology viewpoint.

**Figure 2:**
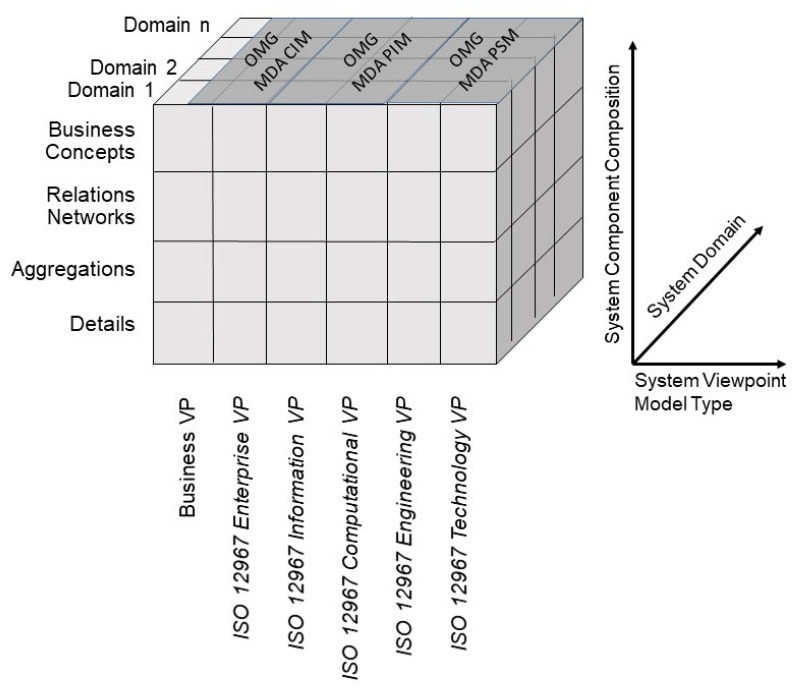
Generic reference architecture.

ISO 23903 Integration and Interoperability Reference Architecture allows the formal representation, design, and management of any ecosystem. Therefore, it must extend the RM-ODP by inclusion of the real-world system viewpoint, called the business viewpoint (VP). In this way, it also provides a methodology for integration and interoperability of any specifications and work products according to the aforementioned standards families. Figure 2 defines its development process dimension, thereby integrating the two other standards.

The GCM approach has been deployed by the first author previously for the development of standards and projects such as ISO 22600 Privilege Management and Access Control [9] and other security and privacy specifications, as well as for EHR design and integration. The challenge of representing and managing knowledge has also been addressed.

## 3. Modeling Transformed Health Ecosystems

In scientific modeling, we distinguish four dimensions of data modelling: data, information, knowledge, and knowledge space [10]. In this model, the transformation from data to information considers interpretation, meaning, and semantics; the transformation from information to knowledge considers action, structure, and pragmatics; and the transformation from knowledge to knowledge spaces supports reflection, innovation, and collaboration across domains [11]. Another way of classifying models is the data model level [12], ranging from the very-high-level data model representing the ISO 23903 Business Viewpoint, the high-level data model according to the ISO/IEC 10746 Enterprise Viewpoint, the logical data model level corresponding to the ISO/IEC 10746 Information Viewpoint and Computational Viewpoint, and finally the physical data model level corresponding to the ISO/IEC 10746 Engineering Viewpoint. Furthermore, we can classify data models according to the related information model level [13]. In this classification, we distinguish between external (business viewpoint), conceptual (enterprise viewpoint), logical (information viewpoint and computational viewpoint) and physical (engineering viewpoint). Table 2 summarizes these model classifications.

In the following section, we will consider the described aspects in more detail.

### 3.1. Knowledge Representation

From the modeling perspective, three levels of knowledge representation are distinguished and must be consecutively processed:

(a) Epistemological level (domain-specific modeling);
(b) Notation level (formalization, concept representation);
(c) Processing level (computational, implementations).

A model can therefore be defined as a representation of the objects, properties, relations, and interactions of a domain, enabling rational and active business in the represented domain.

The generalization of domain-specific epistemological models requires their transformation into a universal KR notation. The outcome must be validated in the real-world system and thereafter adopted if needed [14].

### 3.2. Language Aspect of Knowledge Representation

Symbols, operators, and interpretation theory give sequences of symbols meaning within a KR.

A key parameter in choosing or creating a KR is its expressivity. The more expressive a KR, the easier and more compact it is to express a fact or element of knowledge within the semantics and grammar of that KR, and the more compact this expression may be. However, more expressive languages are likely to require more complex logic and algorithms to construct equivalent inferences. A highly expressive KR is also less likely to be complete and decidable. Less expressive KRs may be both complete and decidable [15].

Any business system can be represented using ICT data ontologies. However, the justification of correctness and completeness of the structure and behavior of the represented ecosystem can only be provided from the ecosystem’s business viewpoint using the involved domains’ ontologies. Justification of structure and behavior representation includes the representational components, their underlying concepts, and their relations, but also the related constraints.

**Figure 3:**
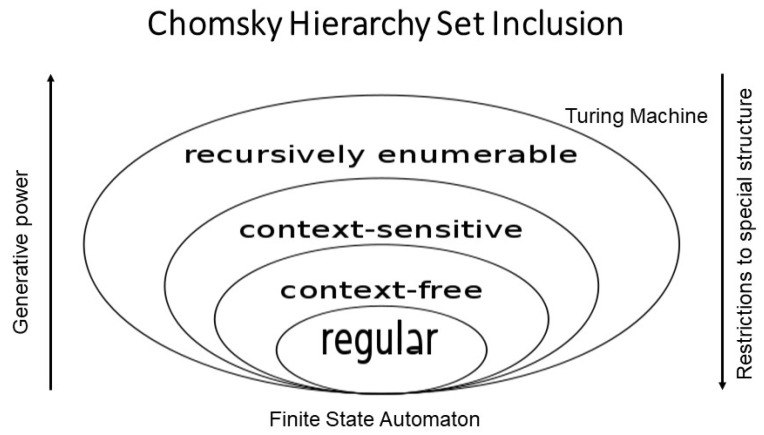
Chomsky Hierarchy [16].

Therefore, natural languages are not only efficient in representing meaning, shared knowledge, skills, and experiences assumed. They also provide an optimum between restriction to special structure and generative power, according to the Chomsky grammar hierarchy (regular, context-free, context-sensitive, recursively enumerable), enabling the rich and nevertheless decidable representation of real-world concepts, supported of course by common-sense knowledge (Figure 3).

**Figure 4:**
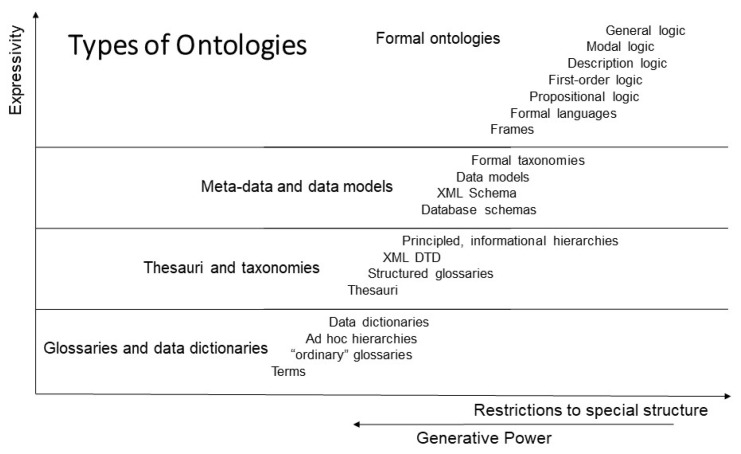
Types of ontologies (from [15], changed).

**Figure 5:**
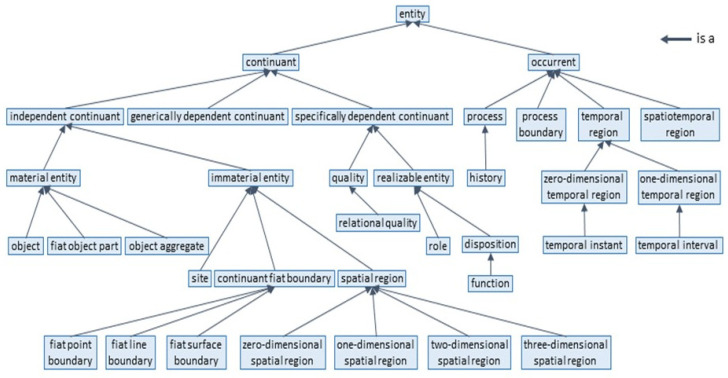
BFO 2020 as a Hierarchy (from [17]).

Knowledge can be represented at different levels of abstraction and expressivity, ranging from implicit knowledge (tacit knowledge) up to fully explicit knowledge representation, i.e., from natural language up to universal logic. Figure 4 presents the different types of ontology. In the event that an ontology is not available, we can deploy as a first step the adopted Top-Level Ontology framework according to ISO/IEC 21838 instead (Figure 5).

### 3.3. Good Modeling Practice (Based on Lankhorst et al. [13])

A model is an unambiguous, abstract conception of some parts or aspects of the real world corresponding to the modeling goals. In this, the domain of discourse, the business objectives, and the stakeholders involved have to be defined.

The corresponding domain experts define the provided view of the model as well as the way of structuring and naming the concepts of the problem space.

Data modeling covers the domain’s concept space, refined to the business concepts, followed by the logical and finally physical models.

**Figure 6:**
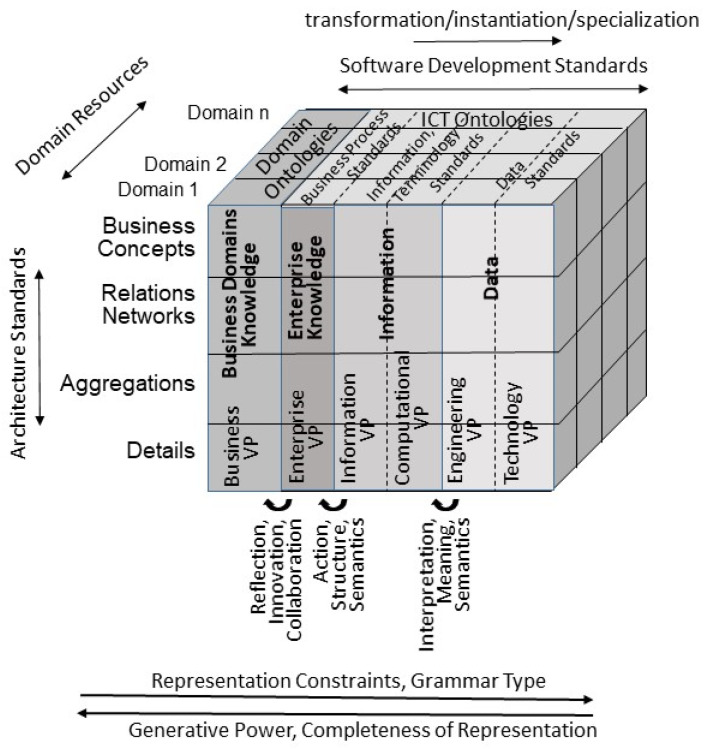
ISO 23903 Framework in the light of good modeling best practices.

After first capturing key concepts and key relations at a high level of abstraction, different abstraction levels should be used iteratively, with the first iteration performed in a top-down manner to guarantee the conceptual integrity of the model. This requires meeting design principles such as orthogonality, generality, parsimony, and propriety. Figure 6 represents the ISO 23903 Framework in the light of good modeling best practices.

**Figure 7:**
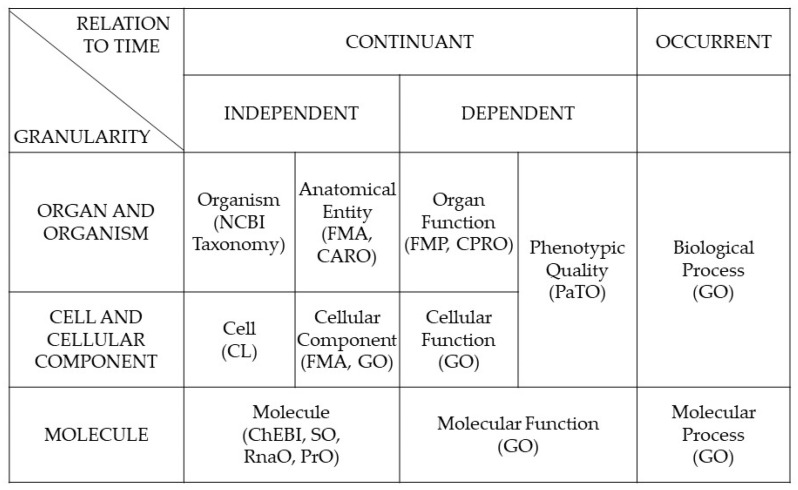
OBO Foundry Ontologies (from [18]).

All views are represented through the related ontologies. Facts as outcomes of observation are represented by data. The interpretation of facts requires information. Understanding the system requires domain concepts (knowledge), and the integration of systems requires external (non-IT) knowledge represented through knowledge spaces. Fact-based practices are realized through observations, resulting in physical data models. Logical data models enable the interpretation of data, while understanding a system requires conceptual data model representation. Managing a real-life system requires external model representation. Figure 7 shows the domain ontologies developed by the Open Biological and Biomedical Ontologies Foundry [18].

To correctly design and manage the transformed health ecosystem, we have to start with the business view represented by domain experts using their terminologies/ontologies. Thereafter, the resulting model must be use case per use case as well as context/constraints per context/constraints transformed in a strict top-down approach into the different views according to the development process. Consequently, the instantiation of the views must be re-engineered according to the knowledge defining the correct and consistent instances, e.g., information and data from studies or repositories, EHRs, biobanks, etc. Table 3 extends Table 1 with the representation styles and related standards.

## 4. Managing Integration and Interoperability for Existing Representational Artifacts

**Figure 8:**
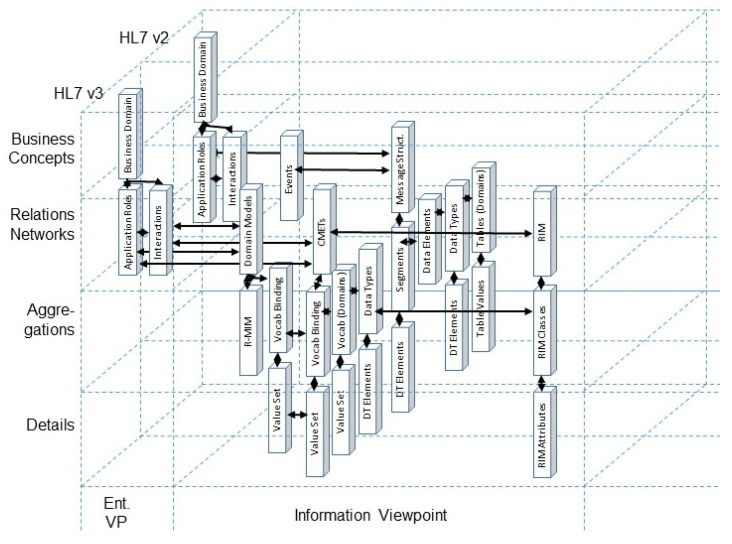
Mapping of HL7 v.2 and HL7 v.3 [7].

**Figure 9:**
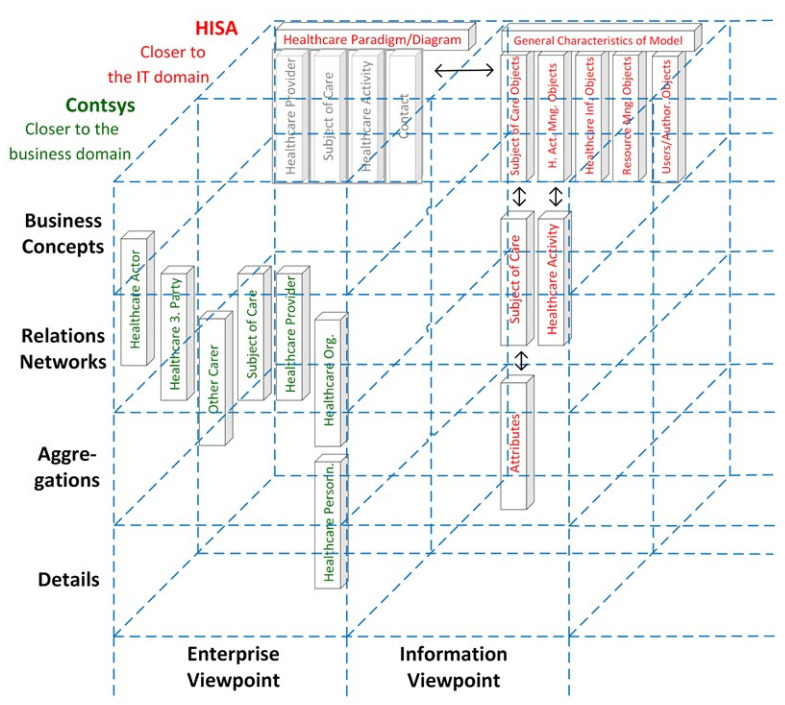
Mapping of ISO 12967 HISA and ISO 13940 ContSys [7].

To manage integration and interoperability for the existing representational artifacts of specific systems, such as standards, specifications or work products, we have to represent them formally according to the model and framework of ISO 23903. This requires the definition of each component and its relations as well as its correct placement regarding the domain, the granularity level, and the view it represents. Thus, we can correctly and consistently represent and interrelate (map) standards, specifications, or work products. Examples are presented in Figure 8 for mapping HL7 v2 and HL7 v3 specifications [19,20,21], and in Figure 9 for mapping ISO 12967 HISA [22] and ISO 13940 ContSys [23].

## 5. Managing Integration of Existing Representational Artifacts into Intended Business Systems for Specific Use Cases

**Figure 10:**
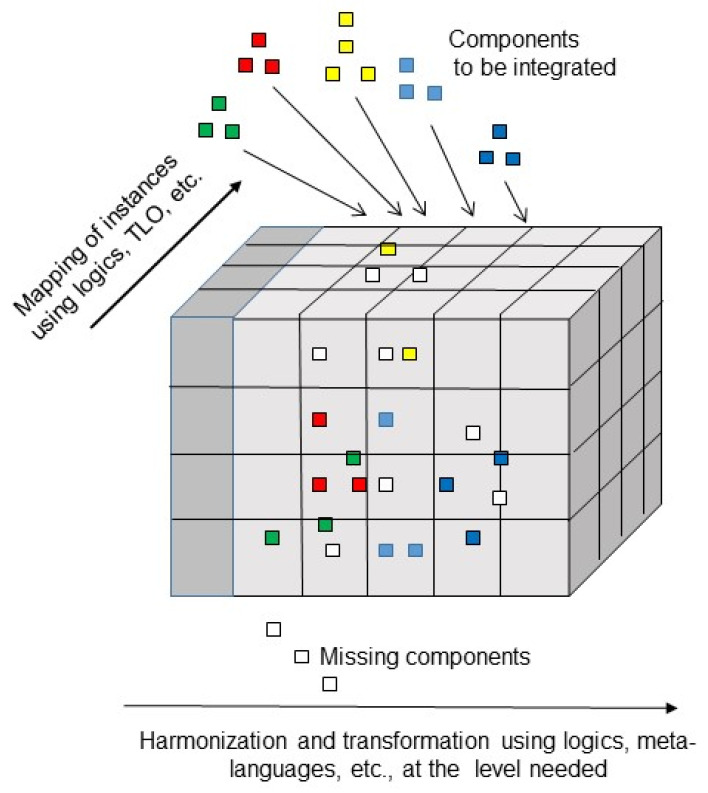
Integration of existing components and representational artifacts.

To integrate different existing representational artifacts in the intended ecosystem, we must first define the use case specific business system architecture regarding structure, function, and relations of its elements/components. Therefore, we have to consider domain- and actor-specific objectives, contexts, constraints, etc. The business system, its components, and relations, forming the business view according to the ISO 23903 model, must be represented using the related domain ontologies. The relations are represented using universal logics. If a domain ontology does not exist, we can adopt the ISO/IEC 21838 Top-Level Ontologies (TLO) to construct a specific ontology such as a patient’s street language representation of his/her problems and expectations, etc., in his/her understanding. The resulting integrated and interoperable model must then be transformed into the different ISO 23903 views up to implementable solutions. The view components can then be instantiated by existing artifacts such as existing process models, information models, FHIR resources, data instances, etc., representing them correctly regarding the domain, the granularity level, and relationships, but also the components’ functions/operations in the context of the use case. To achieve this, we have to re-engineer them according to the ISO 23903 reference model and framework (Figure 10).

**Figure 11:**
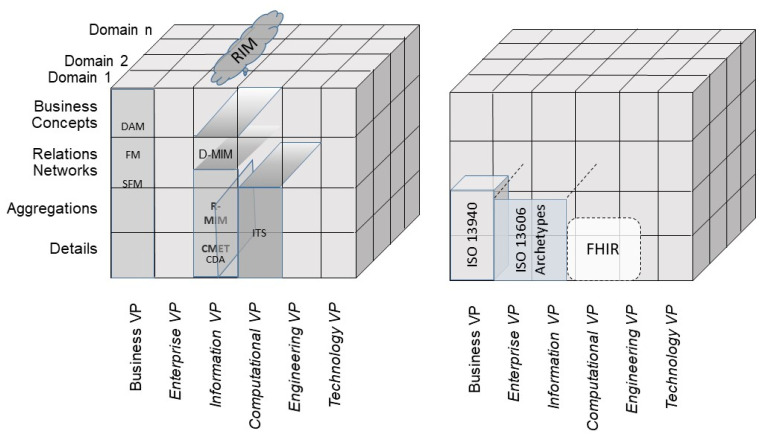
Examples of reusable models.

Possible standard models to be reused are shown in Figure 11, with examples from HL7 and ISO/TC215.

Further examples are medical databases such as EHRs, registries, study protocols, or biobanks. The reused components must be adopted in a manner that is use-case- and concept-specific regarding attributes, relations, etc. In the following sub-chapters, we address the management and mapping of the representational artifacts of software development standards presented in Figure 6, i.e., business process components representing enterprise knowledge, information and terminology standards capturing information, and data representation standards. Finally, we consider the control of the ecosystem’s behavior with a special focus on privacy.

### 5.1. Practical Examples for the Integration of Existing Representational Artifacts

#### 5.1.1. Problems with Mapping Data Representing Different Concepts

**Figure 12:**
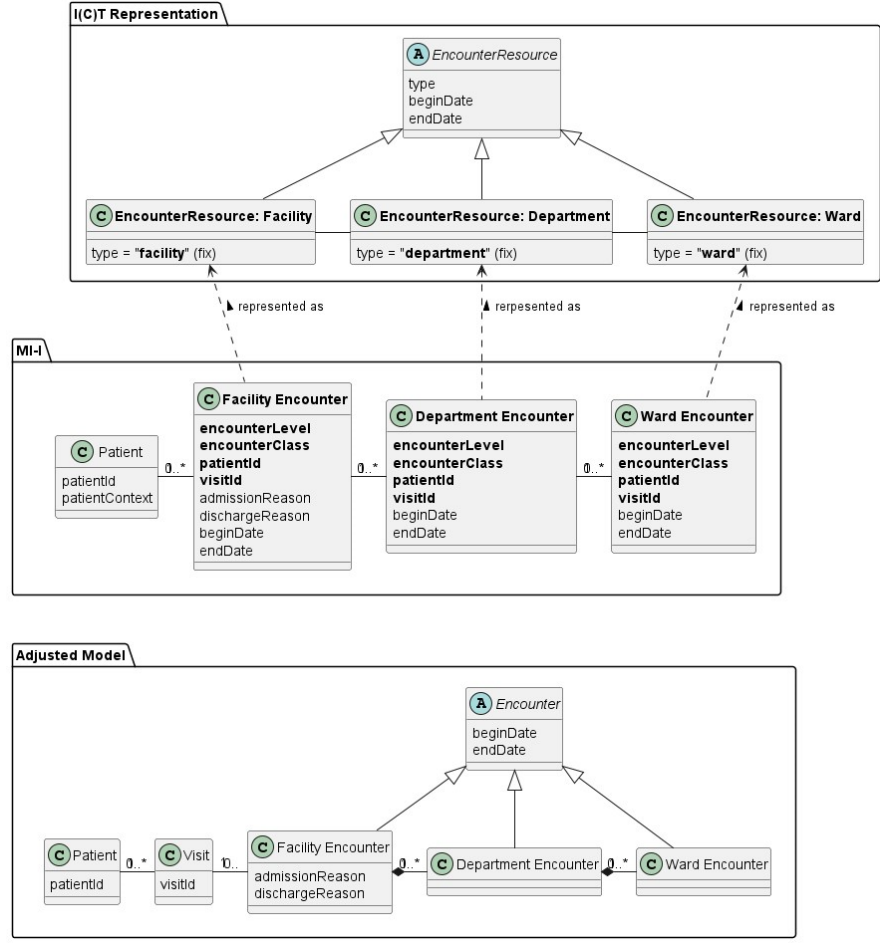
Example model with updates (simplified excerpt from MI-I [24]). * stands for “many” in the sense maxi-cardinality.

The biggest mistake when starting to develop implementation guides for data exchange is to create technological artifacts without an underpinning model. In this case, only profiles to base standards are described, and without any documentation of internal relationships or references to real-world data—business VP as explained above. During the past few years, a considerable number of initiatives have realized that this approach is insufficient and error-prone, and have therefore added several “models”. Unfortunately, these models neither follow a paradigm nor are internally consistent. Figure 12 presents a real-world example that reveals the problems:

The class diagram in the middle is intended to represent an encounter hierarchy that can be found in hospitals: a patient is admitted to hospital (=facility). This includes an association to a department (i.e., specialty) and a nursing unit (ward). In this representation, the classes also contain duplicate information (patient and visit identifier) and redundant details: the classes themselves denote the encounter level. (References to organisational units are left out for simplicity.)

The model in the bottom line is an updated version which only considers the necessary details in a reasonable position, although admission/discharge reason could also be associated with the visit itself. (Both ways have pros and cons.) However, it is much cleaner and easier to read, in addition to offering internal consistency.

The model at the top concentrates on the three distinct variations of encounter (resources/types). Technically, the distinction has been applied by constraining the “type” attribute to a specific fixed value (code). Syntactically, all three forms are identical, but the value/code of the type attribute denotes what this instance represents.

A secondary aspect of this example is the granularity of the items (here, classes) that determine what a specific detail is associated with. This highly impacts translations into proprietary information models, which are created and maintained by the different vendors.

When the artefacts from this example are placed into the aforementioned reference architecture, the bottom model belongs to the information VP, whereas the top model belongs to the technology VP. A transformation between the two with no losses must be possible.

#### 5.1.2. Managing Data Integration in Trauma Centers

Designated trauma centers are a core means of improving trauma care in the US healthcare system. Trauma Institutional Priorities and Teams for Outcomes Efficacy (TIPTOE) is an NIH-funded project (R01GM111324) aiming to fill the knowledge gap regarding whether and how the organizational structures of Level 1 and Level 2 trauma centers affect patient outcomes. In addition, TIPTOE develops a graphical user interface that allows trauma center stakeholders to explore the dependencies between the organizational parameters of their institution and the patient outcomes they generate. Our business case is to visually represent the relationships between organizational parameters and patient outcomes. To implement this idea in a graphical user interface, we need to integrate information from multiple different domains, namely the resource domain, the policy domain, and the medical domain (Table 4).

In the statistical analyses for the TIPTOE project, we concentrate on the following three outcomes: mortality, length of stay, and major complications. Major complications include but are not limited to acute kidney injury, myocardial infarction, pulmonary embolism, severe sepsis, and unplanned admission to the ICU. The data on organizational parameters are collected with a survey tool specifically created for the TIPTOE project [29]. The TIPTOE project recruits Level 1 and Level 2 trauma centers that agree to fill in the TIPTOE survey. In addition to this, they also provide TIPTOE with their Trauma Quality Improvement Program (TQIP) data. TQIP is a program of the American College of Surgeons focusing on elevating the quality of care for trauma patients and collecting data about trauma center performance [30]. TIPTOE derives its patient outcome information from the TQIP data provided by the participating trauma centers. In this specific case, we need to integrate data from the medical domain (patient outcomes) with data from the resource domain, in a way that is consistent with the policy domain.

**Figure 13:**
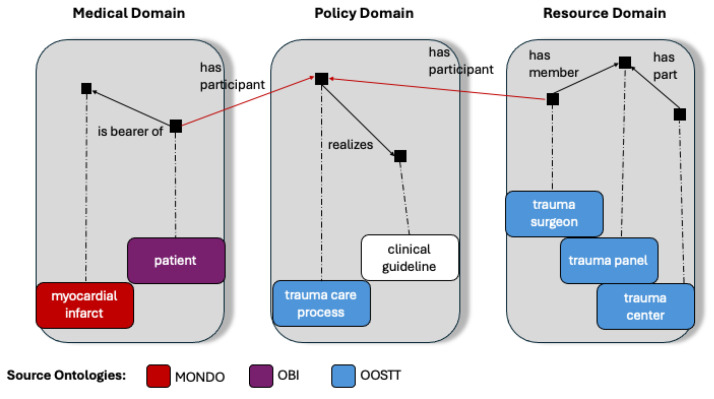
Representation of business view integrating multiple domains. Black squares represent individuals; boxes represent ontology classes; dotted lines link individuals to their classes; and arrows represent relationships between individuals.

Figure 13 shows a practical example of how data is integrated using ISO 23903. Modelers might be tempted to link patient outcomes to each individual trauma center immediately, but that does not correctly reflect the use case. The data collected on organizational parameters are indeed data about features of the trauma center. However, TQIP data are collected on the level of each individual patient in the trauma center. Thus, we need to integrate the different levels in a meaningful way. In selecting the ontologies for this process, we require that the ontologies follow similar design and management principles. This is why we are using OBO Foundry ontologies [31,32] exclusively for this process. The example uses classes and object properties (relations) from the Ontology of Organizational Structures of Trauma Centers and Trauma Systems (OOSTT) [26], the Ontology of Biomedical Investigations (OBI) [32], Mondo Disease Ontology (MONDO) [33], and Relations Ontology (RO) [34]. All organizational features of the trauma center are in the resource domain. They are related to each other in different ways. The patient and their medical condition are in the medical domain. Between these two domains we have the policy domain, which contains the clinical guidelines and their realization in the clinical process, in our case, a trauma care process. Elements of the medical domain and the resource domain are linked to the trauma care process in the policy domain by a “has participant” relation.

A few aspects that we are learning from this work are as follows: (a) we currently do not have a best practice representation of a “clinical guideline”; (b) the placement of “trauma care process” in OOSTT raises the question of whether OOSTT would benefit from a clearer commitment to entities in the resource domain alone and whether it would be better to place entities from the policy domain in their own ontology.

#### 5.1.3. Regional Clinical Data Sharing

Clinical data sharing is a fundamental service for improving medical research and patient care, and for reducing health costs. In order to enable Health Information Systems to correctly understand the content of the exchanged data, the management of semantics is a critical and essential aspect that must be considered. In this context, standardized terminologies, universally recognized for a specific application domain, must be adopted to semantically enriched information to be shared. The deployment of those well-defined tools is not sufficient to solve the problem. In clinical practice, each medical department uses its internal terminology that is usually very different from standardized vocabularies.

Our experience started in 2011 in the Liguria region with the setting up of a data-reuse platform to sustain clinical studies for persons living with HIV [35]. The need to collect data from laboratories spread across the whole region made evident the fact that mapping between concepts at the regional level was mandatory. Concepts from local terminologies were encoded in the corresponding LOINC codes through a translation process. This first translation process was possible only with the considerable involvement of both laboratory technical staff and medical informatics experts, but this work proved to be very time-consuming and subject to rapid obsolescence.

**Figure 14:**
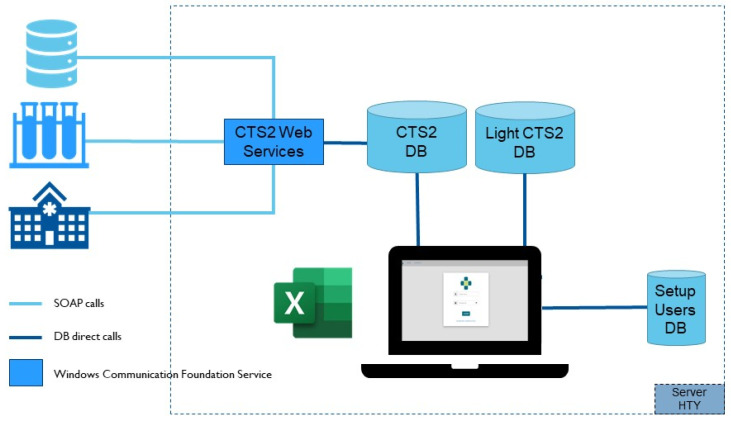
HTS V1 system architecture.

Thanks to a collaboration with a northern Italian region, which requested a system for managing the terminology used by the regional clinical information system, a first prototype of the so-called Health Terminology Service (HTS V0) was developed using a Microsoft SQL database and Windows Communication Foundation Services set (one pair for each local terminology involved), a central Microsoft SQL database, and a web interface. The solution allowed users to manage local terminologies and also map to corresponding standardized vocabulary codes by uploading and downloading the maps through Excel files as required by the final user. In order to maintain the history of the changes in the mapping in an efficient way (due to changes both in local terms and in international vocabulary use), we decided to include in the proposed architecture a tool based on the Common Terminology Service 2 Release 2 (CTS2r2) standard. In particular, for HTS V1, we decided to consider the following as terminology resources: CodeSystem, CodeSystemVersion, EntityDescription, Map, MapVersion, MapEntry; while we considered the following to be functional profiles: Maintenance, Read, Query, History. The Simple Object Access Protocol (SOAP) interfaces as distributed by Health Level 7 (HL7) and by Object Management Group (OMG) were considered implementation technology binding. In our experience, this standard has three key aspects: it is designed by experts in the semantic management field, it is designed to standardize the overall process of vocabulary management, and it provides functionalities to work at atomic level. Using the CTS2 as central element, an architecture was proposed for version 1 of HTS (Figure 14). This was formed by a central relational database based on the CTS2 information model, which stores the terminology content; a database which stores information on the users; a Windows Communication Foundation Service set that represents the CTS2 interface to provide access to the CTS2 database to the external client application; and a user web interface that provides the same functionalities as the V0 version of the web client but interacts with a more complex database. During the implementation, we immediately observed a significant latency in the user experience. For this reason, we decided to add another database which only contained the set of CTS2 data relevant for the final user (the Light CTS2 DB).

The significant latency in the user experience was mainly caused by the very different approaches of the final users (therefore implemented in the web client) and CTS2 in the management of the terminology resources. On the one hand, the final user needs to have an overall overview of the terminologies and the maps, so we presented them in the web client as tables which can be uploaded or downloaded through excel files. On the other hand, as mentioned before, CTS2 was designed to perform operations at atomic level on a single record. So, for example, to show to the user all the codes with their properties of the specific version of a code system, the web client on runtime must obtain the current version of a code system, then the list of the codes, and then resolve each single code to have all its properties. The worst latency problems happen when the user wants to upload a code system formed by tens of thousands of codes via an Excel file. In fact, the solution must perform dynamic check algorithms before updating the single records in the database.

**Figure 15:**
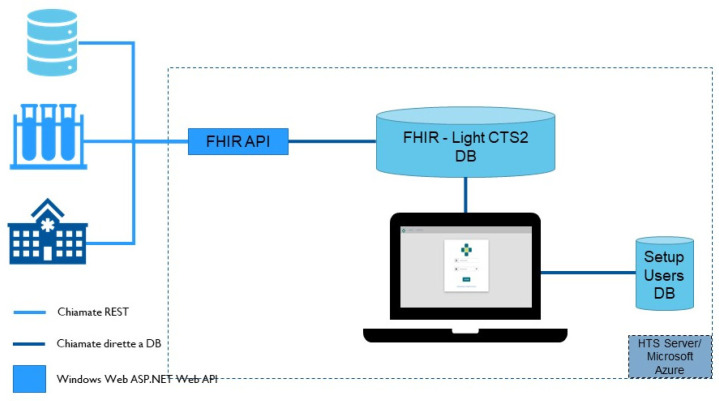
HTS V2 architecture.

In order to improve performance of HTS, a deep re-design phase has been started. First, we considered the FHIR Terminology Server standard that, as with all the FHIR products, is designed to allow faster communication between the applications with a strong focus on implementation. The FHIR specification is less complex than the CTS2 one. Furthermore, HTS performs operations at a macroscopic level on the overall code system or map, so that a unique call can be used to manage the complete transfer of one code system as a whole. Figure 15 shows the HTS V2 system architecture.

This architecture contains both parts of the FHIR terminology system and core elements of the CTS2 system that are missing from the FHIR system.

Operational testing has recently started in many healthcare facilities in the Veneto region. We believe that further improvements will be possible by using the model and framework of the ISO 23903 standard, which supports mapping between ecosystem components at any viewpoint of the system development process. In this way, it allows mapping between local and international terminology systems. We are also evaluating the applicability of natural language processing algorithms in managing updates to this mapping. For this purpose, we have already evaluated the possibility of defining a specific ontology in OWL and correspondingly representing the outcome through the CTS2 standard, published in [36].

#### 5.1.4. Privacy Management in Health Ecosystems

The ability to collect, use, and effectively share multidimensional health data is vital for a successful 5P health ecosystem. The downside of information flow between stakeholders is the increasing possibility for misuse of sensitive personal health information and generation of psychological, social, and economic harms for the data subject/patient. Therefore, to minimize negative consequences to DSs and to society, security and information privacy in health ecosystems should be holistically guaranteed.

The conceptualization and harmonization of privacy is a challenging task. First, privacy is a vague, abstract, cultural, contextual, dynamic concept without a common definition. The ways in which privacy is understood and regulated vary in different cultures and contexts. Privacy as individual right, privacy as control and choice, privacy as legal construct, and risk-based privacy are examples of widely used privacy approaches [37]. Secondly, in the health ecosystem, different stakeholders (e.g., patient, service providers, clinicians, researchers, and regulators) often have their own business goals and objectives concerning the use and sharing of health data as well as its negative consequences. Commonly, privacy needs and rules are also expressed using the stakeholder’s own or local domain ontologies [2].

In different countries, there can be meaningful differences concerning how privacy is understood and regulated, and how health data is defined. Sometimes these differences are even fundamental. For example, the European Union (EU) has a uniform legal privacy framework (the General Data Protection Regulation, GDPR) in which privacy is established as a human right and an individual right to privacy is set out [38]. The European GDPR defines health data as any data concerning health. In the USA, there is no umbrella legislation for privacy. Instead, sectoral fragmented approaches for data protection are used, and the level of protection varies depending on the context [39]. The US Health Insurance Portability and Accountability Act of 1996 (HIPAA) is a federal law that requires the creation of national standards to protect sensitive patient health information from being disclosed without the patient’s consent or knowledge. The concept of “reasonable expectation of privacy” is widely used, and some states have privacy legislation that does not require patient consent prior to the exchange of health information between providers [40,41]. In general, a rights-based privacy approach is the dominant privacy theory nowadays [42]

To enable privacy in real life health ecosystems, it is necessary to answer the previously discussed challenges holistically. A solution is needed which presents and balances different interests and privacy needs of multiple stakeholders (e.g., data subjects, researchers, sponsors, and society) in the ecosystem [41]. Because, in real life, privacy needs and obligations are typically expressed in the form of policies and rules, language presenting these rights and needs is needed. Furthermore, privacy as a concept should be formalized at domain level and mapped at ecosystem level [37,41]. A legally compliant ontology should be developed, as should a solution to guarantee regulatory compliance at ecosystem level. Developed privacy ontologies should represent privacy terms such as control, risk, harm, misuse, data processor, secondary user, contract, context, data subject, patient, concern, choice, autonomy, and freedom.

During the last decade, researchers have developed some answers to these challenges. In a survey by Esteves et al. [42], 13 different privacy languages, including languages for expressing rights, and 9 data protection languages were discovered. For example, Privacy Ontology (PrOnto) includes agents, roles, legal rules, purposes, and legal bases, and it is aimed at legal reasoning. The Data Protection Ontology (DPO) developed by Bartolini and Muthuri focuses on the obligations of the data controller as stated by the GDPR [43]. LIoPY, developed by Loukil et al., is a new legal-compliant ontology to preserve privacy for the Internet of Things. It uses a common privacy vocabulary to address the privacy requirements in the IoT environment [44]. Majdoabi et al. have developed an ontology (HOPPy) for smart health care. It supports privacy policies, user preferences, and compliance with laws and regulations [45].

These examples show that there are promising candidate privacy models and ontologies, but further development and/or integration is needed to reach an ecosystem-level holistic solution for privacy management.

## 6. Discussion

5PM ecosystems are characterized by advancement from an empirical data-focused to a knowledge-driven concept-focused approach. This requires starting the design and management process with the real-world system by representing multidisciplinary concepts in a comprehensive context, thereby reflecting the knowledge spaces of all the domains and actors involved in the specific use case. Starting with data does not allow correct decisions regarding the components, their structures, functions, and relations. ISO 23903 is a sophisticated, foundational, inter-disciplinary, system-theoretical, ontology-based, and policy-driven approach. It also enables the advancement of security and privacy from the Data Protection Directive 95/46/EC towards the GDPR, i.e., from data to concept and process level. This makes ISO 23903 a universal standard enabling the design and management of any system from any domain in any context, covering living and non-living systems, plants, technologies, etc., at any level of granularity from elementary particles up to the universe. All elements in a specific viewpoint representation used/re-used in a specific use case must be re-engineered according to the real-world cross-domain knowledge representation. The corresponding representational challenge of different viewpoint concepts is discussed in some detail.

The presented approach provides integration and interoperability between any ecosystem components, including the actors, by facilitating ontology-based translation within the individual educational background and skills. It is meanwhile mandatory for all projects at the ISO, CEN, IEEE, and other standard-developing organizations (SDOs), covering more than just one domain.

While related papers focus only on one aspect, perspective, or domain, this paper provides a foundational and comprehensive approach to designing and managing advanced ecosystems. As demonstrated in many keynotes at global events, the approach is not limited to the health and social care domain, but applicable in any technical domain, in social and natural sciences, ecology, linguistics, etc. Future papers will extend the provided perspectives by including other domains, other contexts, etc.

A short version of the paper has been published in the pHealth 2024 Proceedings at IOS Press [46].

## 7. Conclusions

Based on a sophisticated, foundational, system-theoretical, architecture-centric, ontology-based, policy-driven approach, ISO 23903 achieves the following:

- Enables representation and management of multiple domains’ knowledge → Understanding the pathology of diseases and sharing that knowledge;
- Enables the integration of different domains;
- Enables interoperability and integration of existing specifications and artifacts, thereby supporting sustainability;
- Enables interoperability between any ecosystem components including the involved actors;
- Serves as a methodology for developing advanced 5P medicine solutions.

## Figures and Tables

**Table 1:** Health and social care transformation towards 5P medicine.

Care Paradigm	Services	Way of Practicing	Justification
Phenomenological Medicine	Domain-specific general services—one solution fits all	Observation	Pattern recognition
Evidence-Based Medicine	Domain-specific, group-specific services	Observation with objective evaluation	Statistical justification of group-specific treatment outcome
Person-Centered Medicine	Multiple domains’ services	Managed care	Process mgmt., best medical practice guidelines
Personalized Medicine	Multiple domains’ services—Telemedicine	Considering the translational pathology of disease	Clinically justified individual status and context
P5 Medicine	Cross-domain services—Consumerism, Telemedicine	Understanding the pathology of disease from elementary particle up to the society	Scientifically justified individual status

**Table 2:** Classification of models.

Data Model Level	Dimension of Modelling	Data Models at Different Information Levels	Modelling Actors	Model Scope	ISO 23903 Interoperability and Integration Reference Architecture	Examples		
Very high level data model	Knowledge space	External	Business domain stake-holders	Scope, requirements and related basic concepts of business case	Business Viewpoint			ISO 23903 Interoperability and Integration Reference Architecture
High level data model	Knowledge	Conceptual	Business domain stake-holders	Relevant information and representation and relationships of basic concepts	Enterprise Viewpoint	DCM, CSO	ISO 10746 ODP RM [8]	
Logical data model	Information	Logical	Data modelers and analysts	Layout and types of data and object relationships	Information Viewpoint	HL7 v3 (CMETs), HL7 CIMI, openEHR Archetypes, FHIM		
					Computational Viewpoint	HL7 FHIR		
Physical data model	Data	Physical	Data modelers and developers	Information-related and platform-specific aspects	Engineering Viewpoint			
					Technology Viewpoint	HL7 v2/3 ITS, SQL, OHDSI, OMOP		

**Table 3:** Representation styles and related standards in the context of health and social care transformation towards 5P medicine.

Care Paradigm	Services	Way of Practicing	Justification	Representation Style	Electronic Comm./Coop.	Standard
Pheno-menological Medicine	Domain-specific general services, one solutions fits all	Observation	Pattern recognition	Data	Local data repository inside the unit	Data standards
Evidence-Based Medicine	Domain-specific, group-specific services	Observation with objective evaluation	Statistical justification of group-specific treatment outcome	Information	Central data repositories	Information standards
Person-Centered Medicine	Multiple domains’ services	Managed care	Process mgmt., Best Medical Practice Guidelines	Agreed terminology, DMP Best Practice Guidelines	Cross-organiza-tional Business Process	Terminology standard, Process standards
Personalized Medicine	Multiple domains’ services, Telemedicine	Considering the pathology of disease	Clinically justified individual status and context	Disciplinary concepts in situational context	Knowledge management	Domain ontology standards
5P Medicine	Cross-domain services, Consumerism, Telemedicine	Understanding the pathology of disease	Scientifically justified individual status	Multidisciplinary concepts in comprehensive context	Knowledge Space management	Multiple ontologies guided by Top Level Ontologies standards

**Table 4:** Domains of orthopedic trauma care with examples of relevant entities and potential domain ontologies (from [25]).

Domain	Types of Entity in Trauma Care	Potential Ontologies
Resource Domain	Organization: Trauma center, trauma system, emergency medical services (EMS), trauma teamHuman Individual: Trauma patient, trauma medical director, trauma registrar, trauma surgeon, EMS personnel, plastic surgeon, infectiologist, microbiologist, surgical pathologist, endocrinologist, radiologistFacilities: EMS vehicle, emergency room, trauma biobank, trauma registry	OOSTT [26], OMRSE
Policy Domain	Resources for optimal care for injured patients [27]; EAST Practice Management Guidelines [28]; trauma system policies [29]; triage plans; clinical guidelines	
Medical Domain	Specimens: Bone specimen; skin specimen; subcutaneous tissue specimen; small vein specimen, muscle specimenDiagnosis: Osteomyelitis, post-operative arthritis, pseudoarthrosisTreatment: Resection, reconstruction, prosthesis, amputation, chronic injection therapy, nerve blocks for chronic painPathological process: Inflammation, systemic inflammatory response syndrome, infection, necrosis, wound healing, bone regeneration, stress-induced hyperglycemia	OBIB, OBI [30,31,32], SNOMED-CT

## Data Availability

The original contributions presented in the study are included in the article.

## References

[1] Byju’s Structure, Functions, Units and Types of Ecosystem. https://byjus.com/biology.

[2] Blobel B.G.M.E., Kalra D. (2023). Managing Healthcare Transformation Towards P5 Medicine.

[3] (2021). Interoperability and Integration Reference Architecture–Model and Framework.

[4] Gruber T., Liu L., Özsu M.T. (2009). Ontology Definition. The Encyclopedia of Database Systems.

[5] Bloe R., Kamareddine F., Nederpelt R. (1996). The barendregt cube with definitions and generalized reduction. Inf. Comput..

[6] Blobel B., Oemig F., Ruotsalainen P., Lopez D.M. (2022). Transformation of Health and Social Care Systems—An Interdisciplinary Approach toward a Foundational Architecture. Front. Med..

[7] Modellgetriebene Architektur. https://de.wikipedia.org/wiki/Modellgetriebene_Architektur.

[8] (1998). Reference Model for Open Distributed Processing (RM-ODP).

[9] (2014). Privilege Management and Access Control.

[10] Wikipedia: Scientific Modelling. https://en.wikipedia.org/wiki/Scientific_modelling.

[11] Krogstie J. (2011). Business Information Systems Utilizing the Future Internet. Perspectives in Business Informatics Research: 10th International Conference, BIR 2011, Riga, Latvia, 6–8 October 2011.

[12] Jaeger G., Rogers J. (2012). Formal language theory: Refining the Chomsky hierarchy. Philos. Trans. R. Soc. B Biol. Sci..

[13] Lankhorst M. (2009). Enterprise Architecture at Work.

[14] Doerner H., Grabowski J., Jantke K.P., Thiele H. (1989). Knowledge representation. ideas–aspects–formalisms. Foundations of Artificial Intelligence.

[15] Rebstock M., Fengel J., Paulheim H. (2008). Ontologies-Based Business Integration.

[16] Chomsky Hierarchy. https://en.wikipedia.org/wiki/Chomsky_hierarchy.

[17] (2020). Information Technology.

[18] Smith B., Ashburner M., Rosse C., Bard J., Bug W., Ceusters W., Goldberg L.J., Eilbeck K., Ireland A., Mungall C.J. (2007). The OBO Foundry: Coordinated evolution of ontologies to support biomedical data integration. Nat. Biotechnol..

[19] Oemig F., Blobel B. (2011). A formal analysis of HL7 Version 2.x. Stud. Health Technol. Inform..

[20] HL7 International Version 2 Product Suite.

[21] HL7 International Version 3 Product Suite.

[22] (2020). Health Informatics Service Architecture (HISA).

[23] (2015). Health Informatics-System of Concepts to Support Continuity of Care (ContSys).

[24] Medical Informatics Initiative MII IG Fall DE v2024. https://simplifier.net/guide/mii-ig-modul-fall-2024-de?version=current.

[25] Brochhausen M., Whorton J.M., Zayas C.E., Kimbrell M.P., Bost S.J., Singh N., Brochhausen C., Sexton K.W., Blobel B. (2022). Assessing the need for semantic data integration for surgical biobanks—A knowledge representation perspective. J. Pers. Med..

[26] Utecht J., Judkins J., Otte J.N., Colvin T., Rogers N., Rose R., Alvi M., Hicks A., Ball J., Bowman S.M. (2016). OOSTT: A Resource for Analyzing the Organizational Structures of Trauma Centers and Trauma Systems. CEUR Workshop Proceedings.

[27] American College of Surgeons and Committee on Trauma (2014). Resources for Optimal Care of the Injured Patient.

[28] EAST Practice Management Guidelines. https://www.east.org/education-career-development/practice-management-guidelines.

[29] Brochhausen M., Ball J., Sanddal N.D., Dodd J., Braun N., Bost S., Utecht J., Winchell R.J., Sexton K.W. (2020). Collecting Data on Organizational Structures of Trauma Centers: The CAFE Web Service. J. Trauma Surg. Acute Care Open.

[30] Shafi S., Nathens A.B., Cryer H.G., Hemmila M.R., Pasquale M.D., Clark D.E., Neal M., Goble S., Meredith W.J., Fildes J.J. (2009). The Trauma Quality Improvement Program of the American College of Surgeons Committee on Trauma. J. Am. Coll. Surg..

[31] Jackson R., Matentzoglu N., Overton J.A., Vita R., Balhoff J.P., Buttigieg P.L., Carbon S., Courtot M., Diehl A.D., Dooley D.M. (2021). OBO Foundry in 2021: operationalizing open data principles to evaluate ontologies. Database.

[32] Ontology for Biomedical Investigations Community Standard for Scientific Data Integration. https://obi-ontology.org.

[33] Vasilevsky N.A., Matentzoglu N.A., Toro S., Flack IV J.E., Hegde H., Unni D.R., Alyea G.F., Amberger J.S., Babb L., Balhoff J.P. (2022). Mondo: Unifying diseases for the world, by the world. MedRxiv.

[34] https://github.com/oborel/obo-relations.

[35] Mora S., Giannini B., Di Biagio A., Cenderello G., Nicolini L., Taramasso L., Dentone C., Bassetti M., Giacomini M. (2023). Ten years of medical informatics and standards support for Clinical Research in an Infectious Diseases Network. Appl. Clin. Inform..

[36] Blobel B. (2024). Selected Papers from the pHealth 2022 Conference, Oslo, Norway, 8–10 November 2022. J. Pers. Med..

[37] Ruotsalainen P., Blobel B. (2023). Future pHealth Ecosystem-Holistic View on Privacy and Trust. J. Pers. Med..

[38] https://www.edps.europa.eu/data-protection/our-work/subjects/health_en.

[39] Adjerid I., Acquisti A., Telang R., Padman R., Adler-Milstein J. (2016). The Impact of Privacy Regulation and Technology Incentives: The Case of Health Information Exchanges. Manag. Sci..

[40] Waldman A.E. (2018). Privacy as Trust.

[41] Piasecki J., Cheah P.Y. (2022). Ownership of individual-level health data, data sharing, and data governance. BMC Med. Ethics.

[42] Esteves B., Rodríguez-Doncel V. (2024). Analysis of ontologies and policy languages to represent information flows in GDPR. Semant. Web.

[43] Bartolini C., Muthuri R. (2015). Reconciling Data Protection Rights and Obligations: An Ontology of the Forthcoming EU Regulation. Workshop on Language and Semantic Technology for Legal Domain. https://orbilu.uni.lu/handle/10993/21969.

[44] Loukil F., Ghedira C., Boukadi K., Benharkat A.-N. LIoPY: A legal compliant ontology to preserve privacy for the Internet of Things. Proceedings of the 2018 IEEE 42nd Annual Computer Software and Applications Conference (COMPSAC).

[45] El Maidoubi D., Bakkali H., Sadaki S., Lehmid A., Maqour Z. HOPPy: Holistic Ontology for Privacy-Preserving in Smart Healthcare Environment. Proceedings of the 2021 Fifth World Conference on Smart Trends in Systems Security and Sustainability (WorldS4).

[46] Blobel B., Oemig F., Ruotsalainen P., Brochhausen M., Giacomini M., Giacomini M., Blobel B., Veltri P. (2024). The Representational Challenge for Designing and Managing 5P Medicine Ecosystems. pHealth 2024.

